# Towards quantitative analysis of conformational landscapes: benchmarking heterogeneous reconstruction tools in cryo-EM

**DOI:** 10.1063/4.0001019

**Published:** 2025-10-27

**Authors:** Laurel F. Kinman, Andrew V. Grassetti, Maria V. Carreira, Joseph H. Davis

**Affiliations:** 1 UCSF, Department of Biochemistry & Biophysics, San Francisco, CA, USA; 2 MIT, Department of Biology, Cambridge, MA, USA

## Abstract

As a single-molecule approach, cryo- electron microscopy (cryo-EM) offers the possibility of reconstructing in exquisite detail the conformational landscapes occupied by dynamic protein complexes. The potential to quantitatively characterize these landscapes is particularly exciting, as it would allow us to understand how drugs, binding partners, and other experimental and environmental perturbations tune these landscapes to regulate biological function. However, quantitative analysis of conformational landscapes via cryo-EM requires both comprehensive identification of all structural states present in a sample, and high-confidence assignment of individual particles to their corresponding conformational state, two tasks that have proven challenging given the low signal-to-noise ratio of the underlying data. While there has been an emergence of statistical and machine learning - based approaches to process highly heterogeneous cryo-EM datasets, we have lacked methods to benchmark the performance of these approaches on either of these two tasks: real datasets lack annotated ground-truth labels, and simulated datasets with ground-truth labels bear few of the noise features of real data. To address this gap, we developed a series of real cryo-EM datasets with encoded ground-truth heterogeneity. By challenging existing heterogeneous reconstruction software pipelines with these datasets, we show that accurate per-particle conformational label assignment remains challenging. We additionally show that these datasets have substantial potential as tools for benchmarking and iterative improvement of heterogeneous reconstruction algorithms.

